# Supplementary figures and images for: Sterilization efficacy of a warm-air circulation system in a vaporized hydrogen peroxide sterilizer
Source: PLoS One. 2026 May 27;21(5):e0347533. doi: 10.1371/journal.pone.0347533 (PMC13215516; doi:10.1371/journal.pone.0347533)

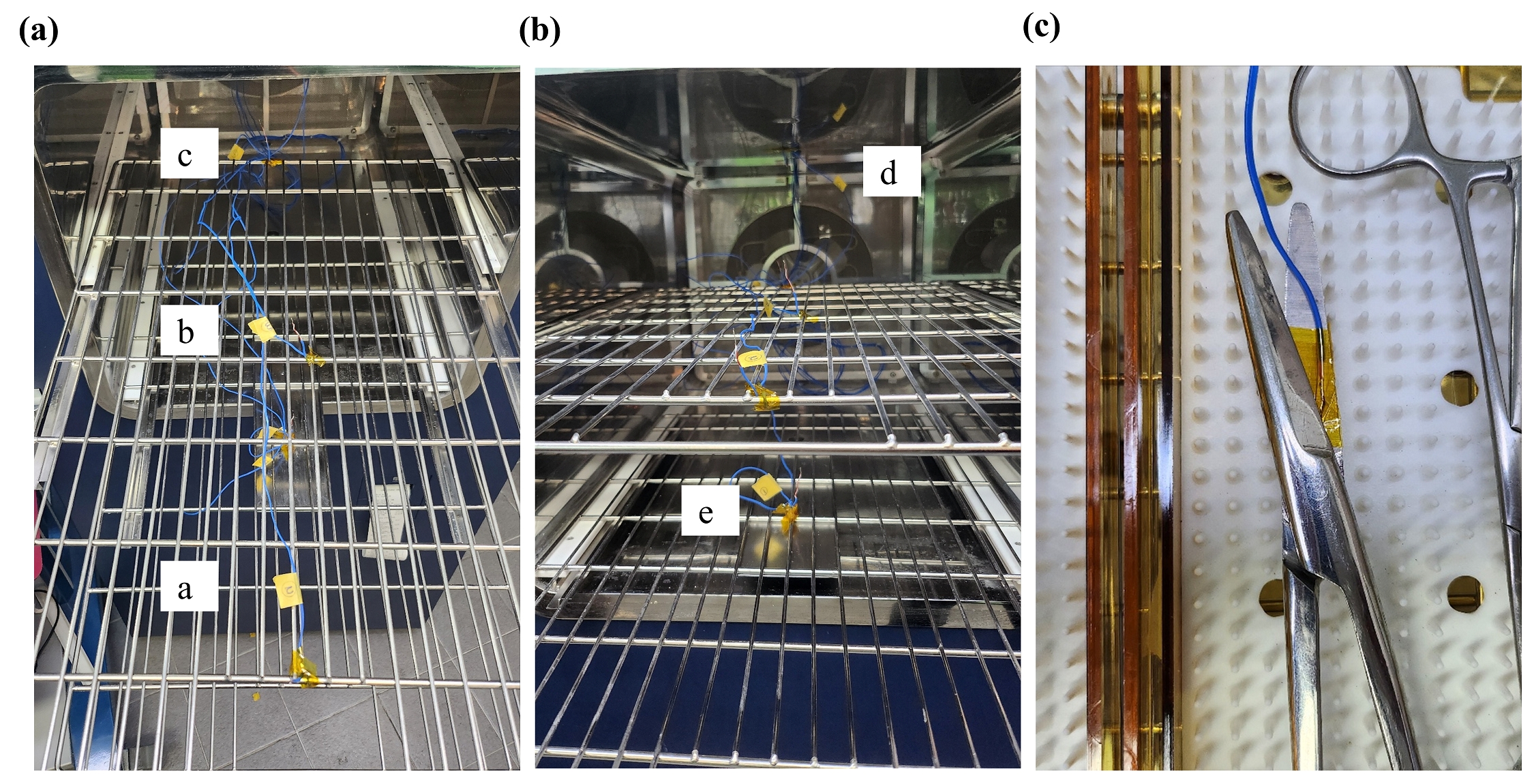

Supplement: S1 Fig — (a) Top view of the chamber (sensor positions: a. front, b. middle, c. rear). (b) Front view of the chamber (sensor positions: d. top, e. bottom). (c) A temperature sensor attached to the hinge surface of surgical scissors within the medical device load. (TIF) [file pone.0347533.s001.tif]

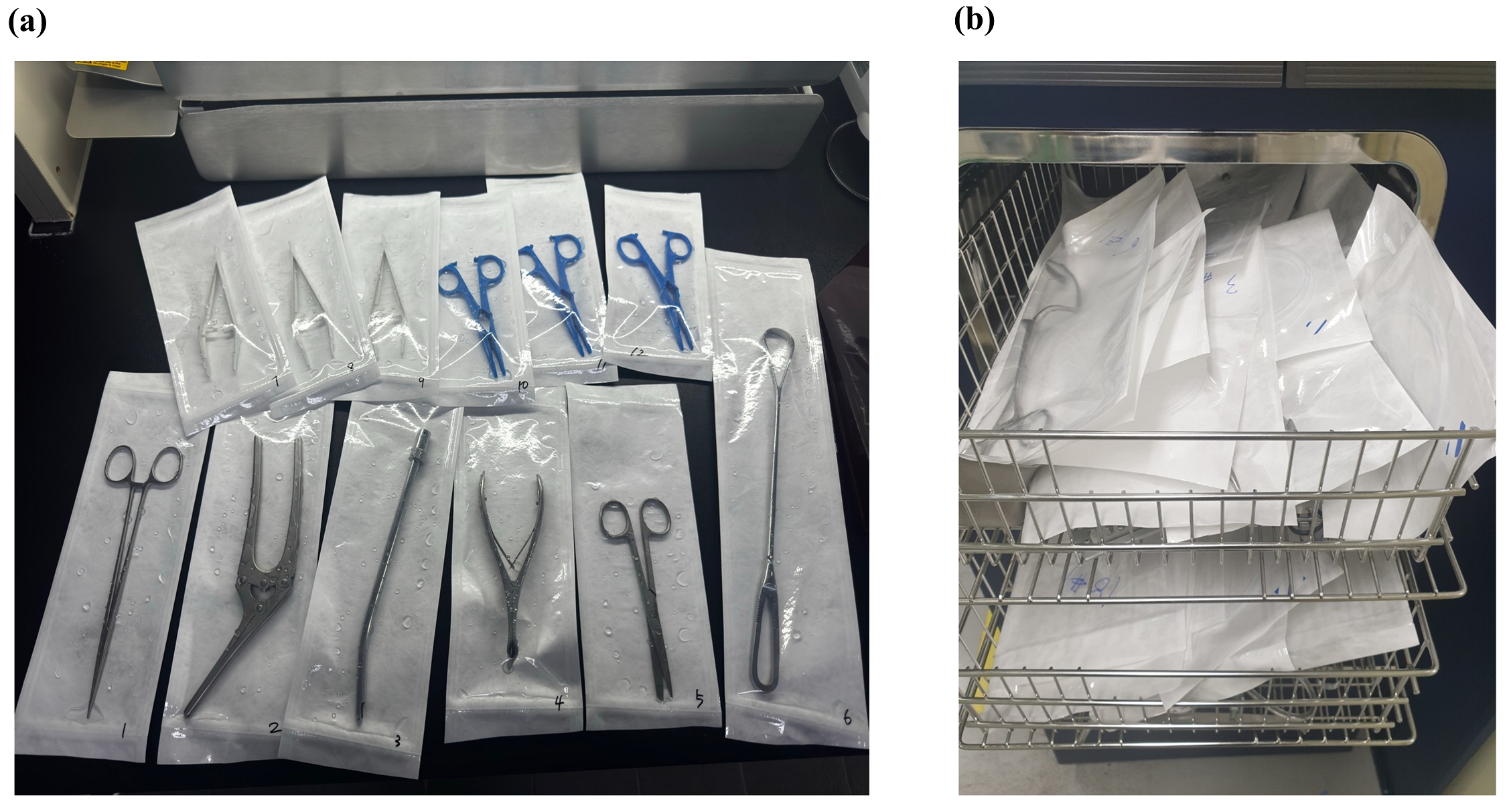

Supplement: S2 Fig — (a) Preparation of representative medical device packages injected with water to simulate wet conditions (see S1 Table for the full list of 24 devices). (b) Configuration of the medical device load inside the sterilization chamber. This setup was used to visually verify moisture reduction efficiency with and without the Warm-Air Circulation System (WACS). (TIF) [file pone.0347533.s002.tif]
