# Supplementary material for: Sterilization efficacy of a warm-air circulation system in a vaporized hydrogen peroxide sterilizer
Source: PLoS One. 2026 May 27;21(5):e0347533. doi: 10.1371/journal.pone.0347533 (PMC13215516; doi:10.1371/journal.pone.0347533)
Supplement: S1 Table — Details include the device name, manufacturer, and material composition. (DOCX) [file pone.0347533.s003.docx]

**S1 table. List of 24 types of medical devices used in the Residual Moisture Removal Test and the Sterilization Efficacy Test.**

| **No.** | **items** | **Manufacturer** | **Material** | **Quantity (n)** |
| --- | --- | --- | --- | --- |
| 1 | Blunt-Curved Scissors | Padgett, USA | Stainless steel | 1 |
| 2 | Nasal Specula | Integra, Germany | Stainless steel | 2 |
| 3 | SP Forceps | Bel-Art, USA | Polypropylene | 3 |
| 4 | Payr Intestinal Clamp | Acheron Instrument, Pakistan | Stainless steel | 2 |
| 5 | Rigid PCD tube | Tri Dente, Australia | Stainless steel | 2 |
| 6 | DE Bakey Forceps | Solco, Korea | Stainless steel | 1 |
| 7 | Vent Filter Set | GSV Filter Technology, UK | Silicon/  Polypropylene | 1 |
| 8 | Cannula | Solco, Korea | Stainless steel | 1 |
| 9 | Rochester-Carmalt Forceps | Jarit, USA | Stainless steel | 1 |
| 10 | Plastic Forceps w/Jaw grips | Mediplast AB, Sweden | Polypropylene | 3 |
| 11 | Suction Tube | Insung Medical, Korea | PVC | 1 |
| 12 | Leksell Rongeur Forceps | V. Mueller, Germany | Stainless Steel | 1 |
| 13 | KIMS Uterine Clamp | Solco, Korea | Stainless steel | 1 |
| 14 | Suction Tube | Insung Medical, Korea | Silicon | 1 |
| 15 | Bone Curette | Ruggles, USA | Stainless steel | 1 |
| 16 | SIMS OBGY Curette | Solco, Korea | Stainless steel | 2 |

Details include the device name, manufacturer, and material composition.
