# Supplementary material for: Sterilization efficacy of a warm-air circulation system in a vaporized hydrogen peroxide sterilizer
Source: PLoS One. 2026 May 27;21(5):e0347533. doi: 10.1371/journal.pone.0347533 (PMC13215516; doi:10.1371/journal.pone.0347533)
Supplement: S2 Table — Data confirms temperature profiles with and without the Warm-Air Circulation System (WACS). (DOCX) [file pone.0347533.s004.docx]

| Warm-air circulation system | Time  (hh:mm:ss) | Bottom | Front | Middle | Rear | Top | Max. Temp.  difference  between locations  (top and bottom) |
| --- | --- | --- | --- | --- | --- | --- | --- |
|  |  | Temperature (°C) | | | | |  |
| **With** | 00:00:00 | 39.2 | 42.1 | 43.1 | 42.9 | 47.5 | 8.3 |
|  | 00:01:00 | 53.1 | 55.5 | 53.5 | 54.0 | 55.3 | 2.4 |
|  | 00:02:00 | 57.8 | 59.4 | 58.0 | 58.3 | 59.7 | 1.9 |
|  | 00:03:00 | 58.1 | 59.5 | 58.2 | 58.3 | 59.3 | 1.4 |
|  | 00:04:00 | 58.0 | 59.3 | 58.1 | 58.0 | 58.7 | 1.3 |
|  | 00:05:00 | 58.0 | 59.0 | 58.0 | 57.9 | 58.4 | 1.1 |
|  | 00:06:00 | 58.0 | 58.9 | 58.1 | 57.9 | 58.4 | 1.0 |
|  | 00:07:00 | 58.0 | 59.0 | 58.1 | 57.9 | 58.3 | 1.1 |
|  | 00:08:00 | 58.1 | 59.0 | 58.1 | 58.0 | 58.3 | 1.0 |
|  | 00:09:00 | 58.2 | 59.0 | 58.2 | 58.1 | 58.4 | 0.9 |
|  | 00:10:00 | 58.2 | 58.9 | 58.2 | 58.1 | 58.4 | 0.8 |
|  | 00:11:00 | 58.1 | 58.7 | 58.2 | 58.0 | 58.3 | 0.7 |
|  | 00:12:00 | 58.1 | 58.6 | 58.1 | 57.9 | 58.2 | 0.7 |
|  | 00:13:00 | 58.2 | 58.7 | 58.2 | 58.1 | 58.3 | 0.6 |
|  | 00:14:00 | 58.2 | 58.7 | 58.2 | 58.1 | 58.3 | 0.6 |
|  | 00:15:00 | 58.3 | 58.7 | 58.3 | 58.2 | 58.4 | 0.5 |
|  | 00:16:00 | 58.3 | 58.8 | 58.3 | 58.3 | 58.5 | 0.5 |
|  | 00:17:00 | 58.4 | 58.7 | 58.3 | 58.3 | 58.5 | 0.4 |
|  | 00:18:00 | 58.4 | 58.7 | 58.4 | 58.3 | 58.5 | 0.4 |
|  | 00:19:00 | 58.3 | 58.6 | 58.3 | 58.2 | 58.4 | 0.4 |
|  | 00:20:00 | 58.3 | 58.6 | 58.2 | 58.2 | 58.3 | 0.4 |
| **Without** | 00:00:00 | 31.5 | 37.0 | 37.8 | 36.4 | 50.9 | 19.4 |
|  | 00:01:00 | 38.9 | 46.5 | 46.8 | 45.5 | 58.4 | 19.5 |
|  | 00:02:00 | 40.3 | 47.8 | 48.2 | 47.2 | 59.2 | 18.9 |
|  | 00:03:00 | 42.1 | 48.5 | 49.0 | 48.3 | 59.9 | 17.8 |
|  | 00:04:00 | 43.3 | 49.1 | 49.8 | 49.3 | 60.3 | 17.0 |
|  | 00:05:00 | 44.4 | 49.6 | 50.3 | 50.0 | 60.5 | 16.1 |
|  | 00:06:00 | 45.2 | 50.3 | 50.9 | 50.5 | 60.5 | 15.3 |
|  | 00:07:00 | 46.0 | 51.1 | 51.3 | 51.0 | 60.8 | 14.8 |
|  | 00:08:00 | 46.7 | 51.3 | 51.8 | 51.4 | 60.9 | 14.2 |
|  | 00:09:00 | 47.4 | 52.1 | 52.0 | 51.7 | 61.1 | 13.7 |
|  | 00:10:00 | 47.9 | 52.0 | 52.3 | 52.0 | 61.1 | 13.2 |
|  | 00:11:00 | 48.2 | 52.6 | 52.6 | 52.3 | 61.3 | 13.1 |
|  | 00:12:00 | 48.7 | 52.6 | 52.9 | 52.5 | 61.4 | 12.7 |
|  | 00:13:00 | 49.0 | 53.0 | 53.2 | 52.8 | 61.4 | 12.4 |
|  | 00:14:00 | 49.4 | 53.3 | 53.4 | 53.0 | 61.6 | 12.2 |
|  | 00:15:00 | 49.7 | 53.3 | 53.8 | 53.3 | 61.8 | 12.1 |
|  | 00:16:00 | 50.0 | 53.5 | 53.9 | 53.5 | 61.9 | 11.9 |
|  | 00:17:00 | 50.2 | 53.7 | 54.1 | 53.7 | 61.8 | 11.6 |
|  | 00:18:00 | 50.4 | 54.0 | 54.2 | 53.9 | 61.8 | 11.4 |
|  | 00:19:00 | 50.6 | 54.2 | 54.4 | 54.1 | 61.8 | 11.2 |
|  | 00:20:00 | 50.9 | 54.4 | 54.7 | 54.3 | 62.0 | 11.1 |

**S2 Table. Comparison of continuous temperature changes by location in the chamber.** Data confirms temperature profiles with and without the WACS.
