# Supplementary material for: Sterilization efficacy of a warm-air circulation system in a vaporized hydrogen peroxide sterilizer
Source: PLoS One. 2026 May 27;21(5):e0347533. doi: 10.1371/journal.pone.0347533 (PMC13215516; doi:10.1371/journal.pone.0347533)
Supplement: S3 Table — Data represents temperature monitoring at the hinge of scissors loaded inside the chamber, with and without the WACS. (DOCX) [file pone.0347533.s005.docx]

| Warm-air circulation system | Time  (hh:mm:ss) | Temperature of  surface of metal scissors (°C) | Temperature increase  over time (°C)  *Cumulative Sum |
| --- | --- | --- | --- |
| **With** | 00:00:00 | 25.7 |  |
|  | 00:01:00 | 29.7 | 4.0 |
|  | 00:02:00 | 33.1 | 7.4 |
|  | 00:03:00 | 36.5 | 10.8 |
|  | 00:04:00 | 40.0 | 14.3 |
|  | 00:05:00 | 42.8 | 17.1 |
|  | 00:06:00 | 45.3 | 19.6 |
|  | 00:07:00 | 47.5 | 21.8 |
|  | 00:08:00 | 49.3 | 23.6 |
|  | 00:09:00 | 50.8 | 25.1 |
|  | 00:10:00 | 52.0 | 26.3 |
|  | 00:11:00 | 53.1 | 27.4 |
|  | 00:12:00 | 53.9 | 28.2 |
|  | 00:13:00 | 54.6 | 28.9 |
|  | 00:14:00 | 55.3 | 29.6 |
|  | 00:15:00 | 55.8 | 30.1 |
|  | 00:16:00 | 56.2 | 30.5 |
|  | 00:17:00 | 56.5 | 30.8 |
|  | 00:18:00 | 56.9 | 31.2 |
|  | 00:19:00 | 57.1 | 31.4 |
|  | 00:20:00 | 57.3 | 31.6 |
| **Without** | 00:00:00 | 24.1 |  |
|  | 00:01:00 | 24.6 | 0.5 |
|  | 00:02:00 | 25.0 | 0.9 |
|  | 00:03:00 | 25.5 | 1.4 |
|  | 00:04:00 | 26.0 | 1.9 |
|  | 00:05:00 | 26.6 | 2.5 |
|  | 00:06:00 | 27.2 | 3.1 |
|  | 00:07:00 | 27.8 | 3.7 |
|  | 00:08:00 | 28.5 | 4.4 |
|  | 00:09:00 | 29.2 | 5.1 |
|  | 00:10:00 | 29.8 | 5.7 |
|  | 00:11:00 | 30.4 | 6.3 |
|  | 00:12:00 | 31.1 | 7.0 |
|  | 00:13:00 | 31.8 | 7.7 |
|  | 00:14:00 | 32.3 | 8.2 |
|  | 00:15:00 | 32.9 | 8.8 |
|  | 00:16:00 | 33.6 | 9.5 |
|  | 00:17:00 | 34.1 | 10.0 |
|  | 00:18:00 | 34.7 | 10.6 |
|  | 00:19:00 | 35.3 | 11.2 |
|  | 00:20:00 | 35.8 | 11.7 |

**S3 Table. Comparison of continuous temperature changes at the scissor hinge.**

Data represents temperature monitoring at the hinge of scissors loaded inside the chamber, with and without the WACS.
