# Supplementary material for: Sterilization efficacy of a warm-air circulation system in a vaporized hydrogen peroxide sterilizer
Source: PLoS One. 2026 May 27;21(5):e0347533. doi: 10.1371/journal.pone.0347533 (PMC13215516; doi:10.1371/journal.pone.0347533)
Supplement: Attachment — Submitted filename: Response to Reviewers_v2.docx [file pone.0347533.s007.docx]

**Response to Reviewers**

- **Manuscript ID:** PONE-D-25-51990
- **Title:** Sterilization efficacy of a warm-air circulation system in a vaporized hydrogen peroxide sterilizer
- **Authors:** Mi Suk Noh, Su Yeon Lee, Eun Gyu Lee, Jonghyun Shin, Sang-Il Lee, Bong-Hyun Jun

**Dear Dr. Shengqian Sun and Reviewers,**

We would like to thank the Academic Editor and the Reviewers for their time and constructive comments on our manuscript. We have carefully reviewed all the suggestions and have revised the manuscript accordingly.

We believe these revisions have significantly improved the quality, clarity, and scientific rigor of our study. Our point-by-point responses to the comments are detailed below.

**Journal Requirements**

**<Requirement 1 (Style Requirements)>**

**Request:** Please ensure that your manuscript meets PLOS ONE's style requirements, including those for file naming.

**Response:** We have carefully reviewed the PLOS ONE style templates and formatted the manuscript accordingly, including file naming conventions and section headings.

**<Requirement 2, 3, 4, 5 (Funding, Role of Funder, and Competing Interests)>**

**Request:**

- Please provide an amended statement that declares *all* the funding or sources of support received during this study.
- Please state what role the funders took in the study.
- Please remove any funding-related text from the manuscript.
- We note that one or more of the authors are employed by a commercial company: LOWTEM Co., Ltd. Please provide an amended Funding Statement declaring this commercial affiliation, as well as an updated Competing Interests Statement.

**Response:** We have **removed the funding information from the Acknowledgments section** of the manuscript as requested. We have also updated the **Funding Statement**, **Role of Funder**, and **Competing Interests Statement** within the cover letter and the online submission system to transparently declare the joint research structure and ensure the objectivity of the study. The specific texts we have entered are as follows:

**1. Funding Statement:** "This study was supported by the Ministry of SMEs and Startups (Grant No. S2634352), South Korea. This study was conducted as a joint research project with LowTem Co., Ltd., which provided the sterilizer, test materials, and salaries for authors **[S.Y.L., E.G.L. and S.I.L.],** and financial support for the external testing fees. There was no additional external funding received for this study."

**2. Role of Funder:** "The funder (LowTem Co., Ltd.) provided support in the form of salaries for authors **[S.Y.L., E.G.L. and S.I.L.]**, provided the sterilizer, manufactured the Process Challenge Devices (PCDs), and covered the costs for the external sterilization efficacy tests conducted by KTC. These authors participated in the conceptualization and study design to verify the device performance. **However, to ensure the objectivity and independence of the results, all sterilization efficacy tests were strictly performed by an external independent agency, the Korea Testing Certification Institute (KTC).** The funder did not have any role in the actual data collection (test execution)."

**3. Competing Interests Statement:** "The authors have read the journal’s policy and the authors of this manuscript have the following competing interests: **[S.Y.L., E.G.L. and S.I.L]** are paid employees of LowTem Co., Ltd. Although LowTem Co., Ltd. provided financial support for the external testing fees, all efficacy data were obtained through an independent third-party laboratory (KTC) to prevent any potential conflict of interest. This commercial affiliation does not alter our adherence to PLOS ONE policies on sharing data and materials. **The other authors have declared that no competing interests exist.**"

**<Requirement 6 (Ethics Statement)>**

**Request:** Please include your full ethics statement in the ‘Methods’ section of your manuscript file. In your statement, please include the full name of the IRB or ethics committee who approved or waived your study, as well as whether or not you obtained informed written or verbal consent.

**Response:** We have added the full ethics statement in the ‘Methods’ section as requested.

**Revised Text:** "This study evaluated the sterilization efficacy of a medical device using commercially available biological indicators and standard test soils. The study did not involve human participants, human biological material, or animal subjects. Therefore, approval from an Institutional Review Board (IRB) or ethics committee was not required."

**<Requirement 7 (Figure 1 Copyright)>**

**Request:** We note that Figure 1 in your submission contain copyrighted images. We require you to either (1) present written permission from the copyright holder to publish these figures specifically under the CC BY 4.0 license, or (2) remove the figures from your submission.

**Response:** We acknowledge the journal’s policy regarding copyright and the Creative Commons Attribution License (CC BY 4.0). To strictly comply with these requirements, **we have replaced the original Figure 1 with a new version.** The updated Figure 1 consists entirely of **original schematics drawn by the authors and/or photographs taken by the authors. We confirm that we hold the full copyright to these images and grant permission for them to be published under the CC BY 4.0 license.** No third-party copyrighted materials are included in the revised figure.

**Revised Text: The figure file for Fig 1 has been replaced with a newly created original image.** (Note: No changes were made to the figure caption text as the image is original.)

(Before)

(After)

**<Requirement 8 (Supporting Information Captions)>**

**Request:** Please include captions for your Supporting Information files at the end of your manuscript, and update any in-text citations to match accordingly.

**Response:** We have complied with the journal's guidelines by adding a dedicated "Supporting information" section at the end of the manuscript, immediately following the References. This section includes the captions for all supporting files. We have also verified that all supporting information files are correctly cited within the main text (e.g., S1 Table).

**Revised Text:**

Supporting Information

**S1 Fig. Location of temperature sensors in the chamber and on medical devices.** (a) Top view of the chamber (sensor positions: a. front, b. middle, c. rear). (b) Front view of the chamber (sensor positions: d. top, e. bottom). (c) A temperature sensor attached to the hinge surface of surgical scissors within the medical device load. **(TIF)**

**S2 Fig. Experimental setup for the residual moisture removal test.** (a) Preparation of **representative** medical device packages injected with water to simulate wet conditions **(see S1 Table for the full list of 24 devices)**. (b) Configuration of the medical device load inside the sterilization chamber. This setup was used to visually verify moisture reduction efficiency with and without the Warm-Air Circulation System (WACS). **(TIF)**

**S1 Table. List of 24 types of medical devices used in the residual moisture removal test and the sterilization efficacy test.** Details include the device name, manufacturer, and material composition. **(DOCX)**

**S2 Table. Comparison of continuous temperature changes by location in the chamber.** Data confirms temperature profiles with and without the WACS. **(DOCX)**

**S3 Table. Comparison of continuous temperature changes at the scissor hinge.** Data represents temperature monitoring at the hinge of scissors loaded inside the chamber, with and without the WACS. **(DOCX)**

**<Requirement 9 (Citation of previous works)>**

**Request:** If the reviewer comments include a recommendation to cite specific previously published works, please review and evaluate these publications to determine whether they are relevant and should be cited. There is no requirement to cite these works unless the editor has indicated otherwise.

**Response:** We have carefully reviewed the reviewers' comments and confirm that there were no recommendations to cite unrelated publications (no citation coercion). However, to address Reviewer #2's request for scientific evidence regarding the spore load, we have **cited relevant standards (e.g., ISO 11138-1) and guidelines** in the revised manuscript.

**Revised Text:** N/A (Relevant citations have been updated in the **Materials and Methods** section and the **References** list.)

**Response to Reviewer #1**

We would like to thank the reviewer for the encouraging comments and the thorough assessment of our manuscript. We have addressed all the major and minor points raised as follows.

**Major points:**

**Comment 1:** "In my view, the efficacy tests (Figs 3 and 4) should include a control with the WACS turned off. For a device used in clinical practice, the expected result would be the inactivation of all test organisms, but without the tests, this cannot be ascertained. This could be an opportunity to demonstrate that efficacy is improved with the WACS."

**Response:** We sincerely appreciate this constructive suggestion. We agree that providing comparative data with a control group (without WACS) is essential to demonstrate the superior efficacy of our system.

To address this, we have **conducted additional comparative tests** and **restructured the manuscript** as follows:

1. **New Data (Table 2):** We performed comparative efficacy tests for cycles **without the WACS** using flexible PCDs and flexible Lumens under residual moisture conditions (10 µL). These tests were conducted across **three independent trials (total n=30 per device type)**.
2. **Restructured Sections:** We have separated the Materials and Methods and Results sections to clearly distinguish between the **comparative efficacy tests under residual moisture conditions** and the **general validation tests for various medical devices**.

The results, now summarized in the newly added **Table 2**, reveal that in the **half-cycles without the WACS**, the **Flexible PCDs resulted in a 100% failure rate (30/30 growth)**, and the **Flexible Lumens showed inconsistent efficacy (8/30 growth)**. In contrast, the **half-cycles with the WACS** achieved **complete inactivation (0/30 growth)** for both device types. This data clearly demonstrates that the WACS is a critical factor in ensuring consistent sterilization efficacy under challenging conditions.

**Revised Text:**

**1. Materials and Methods:** We have added a new subsection titled **"Sterilization efficacy tests for flexible PCDs and flexible lumens with and without the WACS under residual moisture conditions"** to detail the comparative test protocol. Please refer to **Page [9-11], Lines [151-176]** in the revised manuscript for the full description.

**2. Results:** We have restructured the Results section to present the comparative data first (**Page [13], Lines [273-314]**). The key findings are described as follows:

**[Table 2 Caption]** "**Table 2. Comparison of sterilization efficacy for flexible PCDs and lumens with and without the WACS under residual moisture conditions.**"

| **WACS** | **Repeat** | **A** | **B** | **PC** | **NC** |
| --- | --- | --- | --- | --- | --- |
| **With**  (A: Flexible PCDs,  B: Flexible lumens) | 1 | 0/10 | 0/10 | 1/1 | 0/1 |
|  | 2 | 0/10 | 0/10 | 1/1 | 0/1 |
|  | 3 | 0/10 | 0/10 | 1/1 | 0/1 |
| **Without**  (A: Flexible PCDs,  B: Flexible lumens) | 1 | 10/10 | 4/10 | 1/1 | 0/1 |
|  | 2 | 10/10 | 2/10 | 1/1 | 0/1 |
|  | 3 | 10/10 | 2/10 | 1/1 | 0/1 |

Data are presented as (No. of positive cultures) / (Total no. of samples). A positive result indicates bacterial growth and signifies a non-sterile condition.

**[Main Body Text]** (**Page [15], Lines [305-310]**)"Specifically, in the **half-cycles without the WACS**, the flexible PCDs exhibited a **100% failure rate**, with bacterial growth observed in all samples (30/30) across three independent trials. The flexible lumens showed inconsistent efficacy, with growth detected in 2 to 4 samples out of 10 in each trial (8/30 growth). In contrast, the **half-cycles with the WACS** successfully achieved **complete inactivation (0/30 growth)** for both flexible PCDs and flexible lumens, demonstrating consistent sterilization efficacy regardless of the device type."

**Comment 2:** "Currently, the manuscript lacks a discussion in the classical sense. You rightly state that effective sterilisation is important and that WACS seems beneficial. But what are the overarching implications? How often are medical devices currently insufficiently sterilised, and how would implementing a WACS change that? Do you expect the rate of healthcare-associated infections to decrease? Using a WACS seems to lead to higher temperatures of the medical devices – could this cause stability issues? Could one reduce the overall temperature and save energie or the sterilization time to save time?"

**Response:** We appreciate this valuable insight. We have significantly expanded the **Discussion** section to address the overarching clinical implications and material stability as requested.

1. **Clinical Implications:** We highlighted that without the WACS, Flexible PCDs showed **100% failure**, whereas the WACS eliminated this inconsistency. Citing **Karimi Estahbanati [9]**, we explained that residual moisture can freeze and block narrow lumens. We emphasized that the WACS actively removes this moisture, providing a critical **"safety margin"** to prevent sterilization failures that could lead to clinical risks.
2. **Material Stability:** We clarified that the WACS operates **below 60°C**, aligning with **ISO 22441 [12]** for low-temperature sterilization. We also referenced **McEvoy and Rowan [10]** to confirm that this profile is fully compatible with heat-sensitive medical polymers and adhesives used in flexible endoscopes.

**Revised Text:** *(Please refer to* ***Page [19-20], Lines [377-405]*** *in the Discussion section)*

“Notably, the comparative tests under residual moisture conditions highlighted the differing levels of challenge presented by the devices. The flexible PCDs resulted in total sterilization failure (100 % growth) without the WACS, whereas the flexible lumens showed partial and inconsistent failures (20–40 % growth). This confirms that the fabricated flexible PCDs effectively simulate a worst-case scenario that is more rigorous than standard lumens. Most importantly, the WACS successfully overcame these challenging conditions, ensuring complete sterility for both the highly resistant PCDs and the variable lumens, thereby eliminating the risk of inconsistent sterilization outcomes caused by residual moisture.

This elimination of inconsistency has profound clinical implications. Previous studies have emphasized that residual moisture in complex channels can freeze during the vacuum phase, blocking narrow lumens and hindering the efficient contact of the sterilant with microorganisms [9]. Consequently, this mechanism directly leads to load non-sterility. By actively removing this residual moisture, the WACS provides a critical 'safety margin,' preventing potential sterilization failures associated with incomplete drying.

Various devices, including complex lumens and endoscopes, achieved complete sterilization even under challenging conditions verified by BIs. Recent reviews indicate that ensuring sterilant penetration into narrow lumens remains a significant challenge for conventional systems, as residual moisture can physically obstruct these pathways [9]. Our results demonstrate that the WACS facilitates effective vapor penetration even in these difficult-to-sterilize geometries, expanding the applicability of VH₂O₂ sterilization to more complex and challenging medical devices.

Finally, regarding material safety, the WACS ensures stability by maintaining the temperature below 60°C. This aligns with the ISO 22441 standard for low-temperature sterilization [12]. As supported by comprehensive reviews, this low-temperature profile is fully compatible with heat-sensitive medical polymers and adhesives used in flexible endoscopes[10]. Thus, the system ensures material compatibility and device stability while delivering robust sterilization efficacy for complex geometries that are typically difficult to sterilize with conventional VH₂O₂ systems.”

**Minor Points:**

**Comment 1:** "You introduced the abbreviation WACS in the abstract, please introduce it once again in the introduction."

**Response:** Thank you for pointing this out. We have defined the abbreviation **"WACS" (Warm-Air Circulation System)** upon its first appearance in the Introduction section to ensure clarity for the readers.

**Revised Text:** *(Please refer to* ***Page [3], Lines [65]*** *in the Introduction section)* "To overcome these challenges, a warm-air circulation system (WACS) was developed, ..."

**Comment 2:** "Please provide a list of the 24 types of medical device used in the supplements. Ideally, include the manufacturer and material information to help identify which devices are compatible with this setup, especially given the increased temperature of the devices."

**Response:** As requested, we have prepared a detailed list of the 24 types of medical devices, including their manufacturer and material composition. This list has been uploaded as **S1 Table** in the Supporting Information.

**S1 table. List of 24 types of medical devices used in the Residual Moisture Removal Test and the Sterilization Efficacy Test.**

| **No.** | **items** | **Manufacturer** | **Material** | **Quantity (n)** |
| --- | --- | --- | --- | --- |
| 1 | Blunt-Curved Scissors | Padgett, USA | Stainless steel | 1 |
| 2 | Nasal Specula | Integra, Germany | Stainless steel | 2 |
| 3 | SP Forceps | Bel-Art, USA | Polypropylene | 3 |
| 4 | Payr Intestinal Clamp | Acheron Instrument, Pakistan | Stainless steel | 2 |
| 5 | Rigid PCD tube | Tri Dente, Australia | Stainless steel | 2 |
| 6 | DE Bakey Forceps | Solco, Korea | Stainless steel | 1 |
| 7 | Vent Filter Set | GSV Filter Technology, UK | Silicon/  Polypropylene | 1 |
| 8 | Cannula | Solco, Korea | Stainless steel | 1 |
| 9 | Rochester-Carmalt Forceps | Jarit, USA | Stainless steel | 1 |
| 10 | Plastic Forceps w/Jaw grips | Mediplast AB, Sweden | Polypropylene | 3 |
| 11 | Suction Tube | Insung Medical, Korea | PVC | 1 |
| 12 | Leksell Rongeur Forceps | V. Mueller, Germany | Stainless Steel | 1 |
| 13 | KIMS Uterine Clamp | Solco, Korea | Stainless steel | 1 |
| 14 | Suction Tube | Insung Medical, Korea | Silicon | 1 |
| 15 | Bone Curette | Ruggles, USA | Stainless steel | 1 |
| 16 | SIMS OBGY Curette | Solco, Korea | Stainless steel | 2 |

Details include the device name, manufacturer, and material composition.

**Comment 3:** "Just to make sure it is not a typo - for G. stearothermophilus you used 59°C and for the biological indicators 58°C?"

**Response:** Thank you for your meticulous review. The incubation temperature for *G. stearothermophilus* biological indicators was maintained within the manufacturer's recommended range. The discrepancy in the text was a typo. We have corrected the manuscript to consistently state the incubation temperature range as **55–60°C**.

**Revised Text:** *(Please refer to* ***Page [9], Lines [164-166]*** *in the Materials and Methods section)* "... transferred to TSB media (Becton, Dickinson and Company, USA), and incubated at **55~60 °C** for 7 days to check for bacterial growth...."

**Comment 4:** "Table S2 is missing."

**Response:** We apologize for any confusion regarding the accessibility of the previous S2 Table. **Please note that due to the addition of a new S1 Table (**List of medical devices used for the experiments in Table 1**), the previous S2 Table has been renumbered as S3 Table in this revised manuscript.** We confirm that **S3 Table** has been verified, correctly uploaded, and cited in the Supporting Information to ensure it is readily available for review.

**S3 Table. Comparison of continuous temperature changes at the scissor hinge.**

| Warm-air circulation system | Time  (hh:mm:ss) | Temperature of  surface of metal scissors (°C) | Temperature increase  over time (°C)  *Cumulative Sum |
| --- | --- | --- | --- |
| **With** | 00:00:00 | 25.7 |  |
|  | 00:01:00 | 29.7 | 4.0 |
|  | 00:02:00 | 33.1 | 7.4 |
|  | 00:03:00 | 36.5 | 10.8 |
|  | 00:04:00 | 40.0 | 14.3 |
|  | 00:05:00 | 42.8 | 17.1 |
|  | 00:06:00 | 45.3 | 19.6 |
|  | 00:07:00 | 47.5 | 21.8 |
|  | 00:08:00 | 49.3 | 23.6 |
|  | 00:09:00 | 50.8 | 25.1 |
|  | 00:10:00 | 52.0 | 26.3 |
|  | 00:11:00 | 53.1 | 27.4 |
|  | 00:12:00 | 53.9 | 28.2 |
|  | 00:13:00 | 54.6 | 28.9 |
|  | 00:14:00 | 55.3 | 29.6 |
|  | 00:15:00 | 55.8 | 30.1 |
|  | 00:16:00 | 56.2 | 30.5 |
|  | 00:17:00 | 56.5 | 30.8 |
|  | 00:18:00 | 56.9 | 31.2 |
|  | 00:19:00 | 57.1 | 31.4 |
|  | 00:20:00 | 57.3 | 31.6 |
| **Without** | 00:00:00 | 24.1 |  |
|  | 00:01:00 | 24.6 | 0.5 |
|  | 00:02:00 | 25.0 | 0.9 |
|  | 00:03:00 | 25.5 | 1.4 |
|  | 00:04:00 | 26.0 | 1.9 |
|  | 00:05:00 | 26.6 | 2.5 |
|  | 00:06:00 | 27.2 | 3.1 |
|  | 00:07:00 | 27.8 | 3.7 |
|  | 00:08:00 | 28.5 | 4.4 |
|  | 00:09:00 | 29.2 | 5.1 |
|  | 00:10:00 | 29.8 | 5.7 |
|  | 00:11:00 | 30.4 | 6.3 |
|  | 00:12:00 | 31.1 | 7.0 |
|  | 00:13:00 | 31.8 | 7.7 |
|  | 00:14:00 | 32.3 | 8.2 |
|  | 00:15:00 | 32.9 | 8.8 |
|  | 00:16:00 | 33.6 | 9.5 |
|  | 00:17:00 | 34.1 | 10.0 |
|  | 00:18:00 | 34.7 | 10.6 |
|  | 00:19:00 | 35.3 | 11.2 |
|  | 00:20:00 | 35.8 | 11.7 |

Data represents temperature monitoring at the hinge of scissors loaded inside the chamber, with and without the WACS.

**Comment 5:** "With the overall benefits of the WACS, it would be interesting to test reduced cycle times with regard to sterilisation efficacy."

**Response:** We agree that optimizing cycle times using the WACS is a promising direction for future research. While the primary focus of this study was to validate the efficacy and reliability under standard cycle conditions to ensure maximum safety margins, we plan to investigate the potential for cycle time reduction in our follow-up studies.

**Response to Reviewer #2**

We sincerely appreciate the reviewer’s constructive critique and valuable insights regarding our experimental design. We have taken your feedback very seriously and have thoroughly addressed each point. This process has allowed us to significantly improve the clarity of our methodology and better explain our adherence to international standards.

**Comment 1:** "Line numbers should be added."

**Response:** We apologize for the inconvenience. We have added continuous line numbers to the revised manuscript to facilitate the review process.

**Comment 2:**

" Preparation of test organisms and carriers

Two types of spores were prepared: (i) glass fiber spore discs (GFTS-06, Crosstex, Englewood, Colorado), 2.7×10^6^ spores per disc, and (ii) stainless steel wires (0.25 mm×60 mm) inoculated with a spore suspension (VSG-07, Crosstex, Englewood, Colorado), 2.9×10^6^ spores per wire.”

"Any reference for the spore load applied for the sterilization efficacy test? Also, the spores' dose-dependent effect should be tested; at least two or three spore loads should be implemented for the test."

**Response:** We sincerely appreciate the reviewer’s insightful comment regarding the analysis of spore susceptibility. We agree that from a fundamental microbiological perspective, investigating dose-dependent effects is valuable for understanding sterilization kinetics.

However, the primary objective of this study was to validate the sterilization efficacy of a medical device system in strict compliance with international industrial standards, specifically the "Overkill Approach" defined in ISO 11138-1 and ISO 22441.

According to these standards, a sterilization process must demonstrate the capability to inactivate a "worst-case" bioburden, defined as a population of at least 1.0×10^6^ spores of the most resistant organism (*G. stearothermophilus*). The rationale behind this approach is that if the process can successfully eliminate this high-resistance population (10^6^), it inherently guarantees the elimination of lower bioburdens, thereby ensuring a Sterility Assurance Level (SAL) of 10^-6^.

Therefore, instead of testing multiple lower spore loads, we focused on verifying complete inactivation at the most challenging 10^6^ spore load to demonstrate robust safety margins. We have clarified this rationale and added the relevant ISO citations in the **Materials and Methods** section.

**Revised Text:** *(Please refer to* ***Page [7], Lines [131-137]*** *in the Materials and Methods section)*

" In the sterilization efficacy test, *Geobacillus stearothermophilus* spores (ATCC 7953) were used as the representative biological indicator. Selected in accordance with ISO 11138-1[15], this organism is highly resistant to vaporized hydrogen peroxide and was inoculated with a population of $\geq$1.0×10^6^ spores to validate a Sterility Assurance Level (SAL) of 10^-6^ as required by ISO 22441. Since *G. stearothermophilus* is identified as the most resistant microorganism to vaporized hydrogen peroxide, confirming its inactivation ensures the effective sterilization of the entire load [1, 10, 12]."

**Comment 3:** "Table 1. There is no need to show each replicate; please present the data in the style of average ± standard deviation. Also, significant differences, a t-test, or LSD should be added for the data comparison. The method should also include statistical analysis."

**Response:** We strictly agree with the reviewer’s suggestion. Since the data in **Table 1** represents quantitative moisture levels, presenting it as mean ± standard deviation (SD) provides a much clearer assessment than showing individual replicates.

Accordingly, we have re-analyzed the raw data from three independent replicates and updated Table 1 to present the mean ± SD. Furthermore, we performed an independent Student’s t-test to verify statistical significance between the groups (With vs. Without WACS). The analysis confirmed a highly significant difference (*p* < 0.001), which has been indicated in the revised table and caption. We have also added a description of this statistical method to the Materials and Methods section.

**Revised Text:**

**1. Materials and Methods:** *(Please refer to* ***Page [10], Lines [209-214]*** *- New subsection added at the end of Materials and Methods section)*

" All measurements were independently repeated three times. Quantitative results were summarized as mean ± standard deviation (SD), and statistical differences between conditions were evaluated using an independent Student’s t-test. A p-value of < 0.05 was considered statistically significant, and highly significant differences (*p* < 0.001) were noted where applicable. All statistical analyses were performed using Microsoft Excel (Microsoft Corporation, Redmond, WA, USA)."

**2. Results** *(Please refer to* ***Page [13], Lines [256-259]*** *in the Results section)*

As shown in Table 1, the weights of the loads were measured before and after the sterilization cycles. The system with WACS achieved a mean moisture reduction of 12.92 ± 0.43 g, which was significantly higher than the 8.10 ± 0.13 g reduction observed without WACS (*p* < 0.001). These results demonstrate that the WACS effectively removes residual moisture from the load.

**3. Table 1 Contents and Caption** *(Please refer to* ***Page [13], Lines [267-271]*** *in the Results section)*

**Table 1. Moisture reduction before and after sterilization process with and without a WACS.**

| WACS Condition | Initial Weight  (g) | Weight after Sterilization (g) | Moisture Reduction (g) |
| --- | --- | --- | --- |
| With | 2006.74 ± 3.46 | 1993.82 ± 3.51 | 12.92 ± 0.43*** |
| Without | 2004.42 ± 4.75 | 1996.32 ± 4.62 | 8.10 ± 0.13 |

Data are presented as mean ± standard deviation (SD) of three independent replicates (*n* = 3). *** Indicates a significant difference compared to the 'Without WACS' group (*p* < 0.001, Student’s t-test).

**Comment 4:** "Fig.3. It is very confusing for the y-axis; the % growth, actual microorganism load should be presented, instead of %. Also, it is not reasonable that all of the growth for the + control shows 100% growth. Dose-dependent effect should be analyzed."

**Response:** We sincerely appreciate this feedback regarding the clarity of the data presentation. We have made significant changes to the revised manuscript to address your concerns:

1. **Data Presentation (Figures→Tables):** We agree that the Y-axis (% growth) in the original graphs could be confusing given the binary nature of the sterilization data. To eliminate this ambiguity and present the actual results more directly, we have **removed the quantitative graphs** and replaced them with **Table 2, Table 3, and Table 4**. These tables now clearly present the raw data as **"(No. of positive cultures) / (Total no. of samples)"**. This format provides a precise and transparent view of the sterilization outcomes for each device type.
2. **Visualization (New Figure 3):** Instead of data graphs, we have completely revised **Figure 3** to visually illustrate the **physical configurations of the tested devices** (flexible PCD, flexible Lumen, and dual-channel flexible endoscope). This allows readers to intuitively understand the challenging geometries of the sterilization targets without being confused by the graphical representation of binary data.
3. **Positive Control:** We have updated the data presentation to explicitly show the specific number of growing samples (e.g., 1/1, 3/3 or 10/10) rather than a generic "100%". We clarified that the growth observed in all control samples is the **mandatory acceptance criterion** for sterilization validation. If a positive control fails to grow, the entire test is considered invalid. Therefore, the observed growth is a validation requirement, not an experimental anomaly.
4. **Dose-dependent Effect:** As detailed in our response to **Comment 2**, we adhered to the ISO-defined "Overkill Approach" using a fixed high-resistance population (10^6)^, which precludes the need for dose-dependent testing in this specific validation context.

**Revised Text:**

(The original graphs have been replaced by a new Figure 3 and Tables 2-4 in the Results section)

**[New Figure 3 Caption]**

*(Please refer to* ***Page [18], Lines [356-363]*** *in the Results section)*

"**Fig 3. Diverse PCDs, flexible endoscope with inserted BIs for sterilization efficacy tests.** (a) Two types of items with residual moisture: flexible PCD (upper: one side open type, 2 mm inner tube×1,500 mm in length) and flexible lumens (lower: straight type, 1 mm inner tube x 2,000 mm in length); (b) Rigid lumen (1 mm inner tube×500 mm in length); (c) PCDs with challenge packs (right side) for sterilization efficacy tests; (d) Dual-channel flexible endoscope with inserted BI discs. was placed inside the chamber (e). "

**[New Data Tables]**

*(Please refer to* ***Page [17], Lines [311-314]*** *in the Results section)*

"**Table 2. Comparison of sterilization efficacy for flexible PCDs and lumens with and without the WACS under residual moisture conditions.**

| **WACS** | **Repeat** | **A** | **B** | **PC** | **NC** |
| --- | --- | --- | --- | --- | --- |
| **With**  (A: Flexible PCDs,  B: Flexible lumens) | 1 | 0/10 | 0/10 | 1/1 | 0/1 |
|  | 2 | 0/10 | 0/10 | 1/1 | 0/1 |
|  | 3 | 0/10 | 0/10 | 1/1 | 0/1 |
| **Without**  (A: Flexible PCDs,  B: Flexible lumens) | 1 | 10/10 | 4/10 | 1/1 | 0/1 |
|  | 2 | 10/10 | 2/10 | 1/1 | 0/1 |
|  | 3 | 10/10 | 2/10 | 1/1 | 0/1 |

Data are presented as (No. of positive cultures) / (Total no. of samples). A positive result indicates bacterial growth and signifies a non-sterile condition."

*(Please refer to* ***Page [17], Lines [339-341]*** *in the Results section)*

"**Table 3. Sterilization efficacy results for rigid lumens and flexible PCDs.**

| **WACS** | **Repeat** | **A** | **B** | **PC** | **NC** |
| --- | --- | --- | --- | --- | --- |
| **Rigid Lumens**  (A: 1mm x 500mm  B: 0.7mm x 500mm) | 1 | 0/10 | 0/10 | 1/1 | 0/1 |
|  | 2 | 0/10 | 0/10 | 1/1 | 0/1 |
|  | 3 | 0/10 | 0/10 | 1/1 | 0/1 |
| **Flexible PCDs**  (A: 1mm x 1,500mm  B: 2mm x 1,500mm) | 1 | 0/10 | 0/10 | 1/1 | 0/1 |
|  | 2 | 0/10 | 0/10 | 1/1 | 0/1 |
|  | 3 | 0/10 | 0/10 | 1/1 | 0/1 |

Data are presented as (No. of positive cultures) / (Total no. of samples). A positive result indicates bacterial growth and signifies a non-sterile condition."

*(Please refer to* ***Page [18], Lines [353-355]*** *in the Results section)*

"**Table 4. Sterilization efficacy results for dual-channel flexible endoscopes.**

| **Types** | **Repeat** | **A** | **B** | **C** | **PC** | **NC** |
| --- | --- | --- | --- | --- | --- | --- |
| **Dual-channel flexible endoscopes**  (A: BI chamber #1,  B: BI chamber #2,  C: BI chamber #3) | 1 | 0/1 | 0/1 | 0/1 | 1/1 | 0/1 |
|  | 2 | 0/1 | 0/1 | 0/1 | 1/1 | 0/1 |
|  | 3 | 0/1 | 0/1 | 0/1 | 1/1 | 0/1 |

Data are presented as (No. of positive cultures) / (Total no. of samples). A positive result indicates bacterial growth and signifies a non-sterile condition."

We hope that the revised manuscript will now be suitable for publication in PLOS ONE.

Sincerely,

**Bong-Hyun Jun**
Department of Bioscience and Biotechnology
Konkuk University, Seoul, Republic of Korea
Email: bjun@konkuk.ac.kr
Corresponding Author
