# Supplementary material for: Sterilization efficacy of a warm-air circulation system in a vaporized hydrogen peroxide sterilizer
Source: PLoS One. 2026 May 27;21(5):e0347533. doi: 10.1371/journal.pone.0347533 (PMC13215516; doi:10.1371/journal.pone.0347533)
Supplement: Attachment — Submitted filename: Response to Reviewers_revision2.docx [file pone.0347533.s008.docx]

**Response to Reviewers**

- **Manuscript ID:** PONE-D-25-51990R1
- **Title:** Sterilization efficacy of a warm-air circulation system in a vaporized hydrogen peroxide sterilizer
- **Authors:** Mi Suk Noh, Su Yeon Lee, Eun Gyu Lee, Jonghyun Shin, Sang-Il Lee, Bong-Hyun Jun

**Dear Academic Editor and Reviewers,**

We would like to thank the Academic Editor and the Reviewers for their time, constructive feedback, and positive evaluation of our revised manuscript (PONE-D-25-51990R1). We are highly encouraged by the comments and have carefully revised the manuscript to address all the points raised by Reviewer #3.

In addition, per the Journal Requirements, we have reviewed our reference list and confirm that it is complete, correct, and contains no retracted papers. **During this review, we have also corrected minor formatting inconsistencies and incomplete citation details (e.g., adding missing article numbers and URLs) to ensure full compliance with the journal's guidelines.**

Below, we provide a point-by-point response to the reviewer’s comments.

**<Response to Reviewer #2>**

**Comment:** The manuscript has been greatly revised. And I have no more questions. Thanks for the authors revising. Cheers,

**Response:** We sincerely appreciate your positive feedback and your time in reviewing our manuscript. Your insightful comments during the previous round significantly improved the quality of our research. Thank you.

**<Response to Reviewer #3>**

**Comment 1:** Line 175: Please check the brackets.

**Response:** We apologize for the oversight. The brackets in the sentence have been corrected in the revised manuscript.

Before: Similarly, stainless steel wires inoculated with 2.2×10^6^ spores/10 µL, True indicating, USA) were inserted into flexible lumens and subjected to sterilization with moisture-loaded items.

**Revised Text:**

Similarly, stainless steel wires inoculated with 2.2×10^6^ spores/10 µL (True indicating, USA) were inserted into flexible lumens and subjected to sterilization with moisture-loaded items.

**Comment 2:** Lines 228-230: Here a conclusion is already drawn. The statement about „consistent sterilisation performance“ cannot be drawn from the so presented data alone. The sentence should be removed.

**Response:** We completely agree with your point. To maintain objectivity in the Results section, we have removed the premature conclusion regarding "consistent sterilization performance" from this paragraph.

**Before**: The results demonstrated that the WACS facilitated uniform temperature distribution across all positions within the chamber, ensuring consistent sterilization performance regardless of location.

**Revised Text:**

The results demonstrated that the WACS facilitated uniform temperature distribution across all positions within the chamber.

**Comment 3:** Line 240: „A higher temperature increases in the items [..] can [..] remove the [..] moisture.“ Please check the grammar of this sentence. I guess the authors meant „A higher temperature increase…“.

**Response:** Thank you for pointing this out. We have corrected the grammatical error as you suggested. The phrase has been revised to: *"A higher temperature increase in the items..."*

**Before**: A higher temperature increase**s** in the items to be sterilized can more effectively remove the remaining moisture.

**Revised Text:**

A higher temperature increase in the items to be sterilized can more effectively remove the remaining moisture.

**Comment 4:** Line 242: The so far presented data does not allow a conclusion about the removal of sterilisation inhibitors. The data only allows statements regarding the heating and temperature uniformity.

**Response:** We completely agree with your assessment. To avoid drawing a direct conclusion about the removal of sterilization inhibitors in the Results section, we have softened the statement. The sentence has been revised to focus on the rapid heating and the creation of a favorable thermal environment, as suggested.

**Before:** Thus, the WACS, which increases the temperature rapidly for the initial 5 minutes, is effective in removing sterilization inhibitors such as condensation of hydrogen peroxide gas.

**Revised Text:** Thus, the WACS, which increases the temperature rapidly for the initial 5 minutes, provides a favorable thermal environment that may help mitigate the potential condensation of hydrogen peroxide gas.

**Comment 5:** Lines 252-255: Please check the grammar of this paragraph/sentence and split the sentence into two or more shorter sentences.

**Response:** Thank you for this helpful suggestion. We agree that the original sentence was overly long and complex, which hindered readability. We have revised the text by splitting it into three shorter, clearer sentences and correcting the grammatical structure to clearly describe the methodology.

**Before:** Residual moisture removal efficacy was confirmed by measuring and comparing the total weight of validation loads consisting of 24 metal and non-metal medical devices (S1 table) packaged in each pouch injected with 0.5 g of distilled water (total 12 g of moisture) before and after the sterilizer by Special Full cycle with the WACS.

**Revised Text:** Residual moisture removal efficacy was confirmed by measuring and comparing the total weight of validation loads before and after sterilization. The validation loads consisted of 24 metal and non-metal medical devices (S1 Table), each packaged in a pouch containing 0.5g of distilled water (total moisture: 12g). The prepared loads were processed using the Special Full cycle with the WACS.

**Comment 6:** Table 1: With the WACS, 12.92 g of water were removed, but only 12 g of water were added before the WACS-sterilisation. How do the authors explain the additions removal of 0.92 g?

**Response:** Thank you for this insightful question. Although 12 g of distilled water were intentionally introduced, the additional removal of approximately 0.92 g can be attributed to the intrinsic residual moisture inherently present within the complex internal structures (e.g., lumens, crevices) of the medical devices, as well as ambient humidity absorbed before the test. Therefore, the WACS effectively removed not only the intentionally injected 12 g of water but also this pre-existing moisture. To prevent any confusion, we have added a brief explanation to the corresponding paragraph in the revised manuscript. Additionally, to maintain the objectivity of the Results section, we replaced the concluding statement with this factual explanation.

**Before:** As shown in Table 1, the weights of the loads were measured before and after the sterilization cycles. The system with WACS achieved a mean moisture reduction of 12.92 ± 0.43 g, which was significantly higher than the 8.10 ± 0.13 g reduction observed without WACS (p < 0.001). These results demonstrate that the WACS effectively removes residual moisture from the load.

**Revised Text:** As shown in Table 1, the weights of the loads were measured before and after the sterilization cycles. The system with WACS achieved a mean moisture reduction of 12.92 ± 0.43 g, which was significantly higher than the 8.10 ± 0.13 g reduction observed without WACS (p < 0.001). The total weight reduction of 12.92 g slightly exceeded the 12 g of intentionally injected water, which is likely attributable to the evaporation of intrinsic residual moisture initially present within the complex internal structures of the validation loads.

**Comment 7:** Lines 264-266: „This demonstrates that the system effectively eliminates moisture from sterilized items and prevents issues like incomplete drying, condensation, and potential sterilization failure.” This statement is a conclusion and should not be made in the Results part of the manuscript.

**Response:** We fully agree with your comment. To strictly maintain the objectivity of the Results section, we have deleted this concluding statement from the manuscript. Interpretations regarding the system's effectiveness and the prevention of issues such as condensation are exclusively addressed in the Discussion section.

**Before:** The WACS completely removed added moisture from the pouched loads during sterilization cycles, leaving no visible residue. This demonstrates that the system effectively eliminates moisture from sterilized items and prevents issues like incomplete drying, condensation, and potential sterilization failure.

**Revised Text:** The WACS completely removed added moisture from the pouched loads during sterilization cycles, leaving no visible residue.

**Comment 8:** Line 301: „after sterilisation half-cycle” is written twice in the sentence

**Response:** We apologize for this oversight. The duplicated phrase has been removed to correct the sentence.

**Before:** (ii) The stainless-steel wires inoculated with *G. stearothermophilus* spores were inserted into 10 flexible lumens of 1 mm×2,000 mm loaded in two sterilization trays, including 11 kg of medical devices with added moisture, and were found to have positive growth in 2 to 4 out of 10 replicates after sterilization half-cycles after sterilization half-cycles.

**Revised Text:** (ii) The stainless-steel wires inoculated with *G. stearothermophilus* spores were inserted into 10 flexible lumens of 1 mm×2,000 mm loaded in two sterilization trays, including 11 kg of medical devices with added moisture, and were found to have positive growth in 2 to 4 out of 10 replicates after sterilization half-cycles.

**Comment 9:** Line 363: There is a full stop set wrongly in the figure text.

**Response:** We apologize for this typographical error. The incorrectly placed full stop in the figure caption has been removed to ensure the sentence flows correctly.

**Before:** (d) Dual-channel flexible endoscope with inserted BI discs. was placed inside the chamber (e).

**Revised Text:** (d) Dual-channel flexible endoscope with inserted BI discs placed inside the chamber (e).

We believe that the manuscript has been substantially improved thanks to your valuable comments. We hope that the revised manuscript is now suitable for publication in *PLOS ONE*.

Sincerely,

**Bong-Hyun Jun**
Department of Bioscience and Biotechnology
Konkuk University, Seoul, Republic of Korea
Email: bjun@konkuk.ac.kr
Corresponding Author
